# Editorial: Metal Transport in Plants

**DOI:** 10.3389/fpls.2021.644960

**Published:** 2021-02-26

**Authors:** Stephan Clemens, Seckin Eroglu, Louis Grillet, Tomoko Nozoye

**Affiliations:** ^1^Department of Plant Physiology, University of Bayreuth, Bayreuth, Germany; ^2^Department of Biological Sciences, Middle East Technical University, Ankara, Turkey; ^3^Department of Agricultural Chemistry, National Taiwan University, Taipei, Taiwan; ^4^Center for Liberal Arts, Meiji Gakuin University, Tokyo, Japan; ^5^Graduate School of Agricultural and Life Sciences, The University of Tokyo, Tokyo, Japan

**Keywords:** metal, transport, translocation, plant, essential nutrient elements, toxic metal

Plants have to acquire 17 different essential elements (nitrogen, phosphorus, potassium, oxygen, hydrogen, carbon, calcium, magnesium, sulfur, iron, manganese, boron, zinc, molybdenum, copper, chlorine, and nickel) mainly from the rhizosphere by their roots to complete their life cycle. Both shortage and excess of these lead to impaired growth, which is often reflected as yield loss in agriculture. Plants have developed sophisticated and tightly regulated mechanisms for uptake and translocation of the essential elements. On the other hand, plants also take up harmful elements such as cadmium and arsenic non-specifically which may interfere with essential nutrients' uptake. Recent studies have shown that molecular networks to maintain homeostasis of the essential elements are intertwined. How elements are interacting with each other during acquisition and translocation determines yield quality and quantity in agriculture, yet is poorly understood. In this Research Topic, we aim at covering essential nutrient homeostasis in plants; including, but not limited to their transport, translocation and interactions.

The Research Topic succeeded in collecting six original research papers, one review, one perspective and one mini review, which discuss not only the essential but also the toxic metals under various physiological conditions. Escudero et al. reported that iron transport to rhizobia is important for nitrogen fixation. Symbiotic nitrogen fixation by rhizobia in roots of legumes requires relatively large amounts of transition metals. Escudero et al. focused on the model legume *Medicago truncatula*, searched for metal-related symbiotic phenotypes of mutants with defects in symbiosis establishment, and found a mutant whose growth was impaired only under symbiotic condition, since a nicotianamine (NA) synthase gene (*MtNAS2*) was disrupted by insertion of a transposon. NA is known as a chelator involved in iron translocation in plant bodies. Escudero et al. suggested that proper distribution of ferric iron in the nodule is necessary for symbiotic nitrogen fixation.

Ceasar et al. reported on the function of the di-cysteine motifs in the Arabidopsis plasma membrane Heavy Metal ATPase 4 (HMA4) transporter which mediates xylem loading of cadmium for export to shoots, in addition to zinc. The authors revealed that the di-cysteine motifs present in the AtHMA4 C-terminal extension were not essential for cadmium transport from root to shoot, even though the di-cysteine motif had been shown to be essential for high-affinity zinc binding and transport in *planta*. In addition, Ceasar et al. showed that the affinity of the di-cysteine motif was lower for cadmium than for zinc, suggesting that it is possible to identify or engineer HMA4 variants able to discriminate zinc and cadmium transport.

Perea-García et al. investigated the link between copper and iron homeostasis in Arabidopsis. Both copper and iron are transition metals and their reduced forms facilitate the formation of reactive oxygen species (ROS), which can damage plant cells. In some cases, these transition metals can be substituted for one another, in different proteins to perform similar functions. The concentrations of copper and iron are therefore linked to each other. Perea-García et al. analyzed the physiological and transcriptional change in transgenic Arabidopsis overexpressing the high affinity copper transporters COPT1 and COPT3 (COPT^OE^). They found that iron accumulated in roots as copper increased in the media, while iron homeostasis genes were downregulated *in planta*, suggesting that adequate copper uptake is important for iron homeostasis.

A study by Uraguchi et al. showed how gelling agents' contaminants could dramatically influence the elemental accumulation and metal sensitivity in *planta*. Uraguchi et al. used three types of agar and analyzed phenotypes of three established mutants [*Cadmium sensitive (cad)1-3* and *cad1-6*, loss of function alleles of the main phytochelatin (PC) synthase in Arabidopsis, *AtPCS1*; *ATP-binding cassette C* (*abcc)1/2*, the double knockout mutant of *AtABCC1* and *AtABCC2*, Arabidopsis PC-metal (loid) complex transporters on tonoplast]. Uraguchi et al. found that the established phenotypes of these mutants depended on the agar, suggesting that the selection of agar reagents is important and can mask even reliable phenotypes.

Nishida et al. examined two species of nickel hyperaccumulator plants in the genus *Noccaea*: *N. caerulescens* and *N. japonica*. Nishida et al. found that *N. japonica* tolerated excess nickel better than *N. caerulescens*, and the translocation of nickel from root to shoot was lower in *N. japonica* compared to *N. caerulescens*. On the other hand, zinc concentrations in both roots and shoots were similar between *N. japonica* and *N. caerulescens* even under zinc excess conditions. In addition, the expression of the gene encoding the vacuolar nickel transporter *iron-regulated 2* (*IREG2*) was higher in *N. japonica* compared to *N. caerulescens*, suggesting that sequestration of nickel in the vacuole by IREG2 is one reason for the nickel tolerance in *N. japonica*.

Krishna et al. analyzed the sequences of 113 zinc-regulated, iron-regulated transporter-like proteins (ZIP) family from 14 plants species. ZIP family transporters are involved in cellular uptake of zinc, as well as in transport of other divalent metal cations such as cadmium, iron, and copper. Krishna et al. used bioinformatics to show that the plant ZIP transporters are mainly in one cluster together, however some residues involved in metal binding and transport are not conserved among members of the ZIP family, suggesting that distinct metal transport mechanism may exist. It was suggested that manipulation of critical amino acid residues of ZIP transporters may improve zinc homeostasis in planta.

In the review and perspective articles, Rai and Kawabata discuss the mechanisms of cesium uptake into rice plants which has been a serious problem following the accidents at the nuclear power plants of Chernobyl and Fukushima Daiichi. Roschzttardtz et al. summarized the transcriptional regulation of iron distribution in Arabidopsis seeds. Aung and Masuda reviewed recent findings about the molecular mechanisms defending rice plants against excess iron.

The present Research Topic shows that the mechanism of the essential and toxic metals overlap, but are not entirely identical. Results suggest that the manipulation of metal transport systems has the potential to generate high quality crops, with high yield and abundant essential nutrients, while largely excluding toxic metals.

## Author Contributions

TN wrote a draft manuscript. SC, SE, and LG revised the manuscript. All authors contributed to the article and approved the submitted version.

## Conflict of Interest

The authors declare that the research was conducted in the absence of any commercial or financial relationships that could be construed as a potential conflict of interest.

